# The Inventory of Physical Activity Barriers: Development and Preliminary Validation

**DOI:** 10.1093/geroni/igaa057.617

**Published:** 2020-12-16

**Authors:** Mariana Wingood, Nancy Gell, Denise Peters, Tiffany Hutchins

**Affiliations:** 1 University of Vermont, Waterbury, Vermont, United States; 2 The University of Vermont, Burlington, Vermont, United States; 3 University of Vermont, Burlington, Vermont, United States

## Abstract

Inactivity levels among community-dwelling adults 50 years and older is a healthcare concern, particularly when examining the association between increasing age, inactivity, and risk of non-communicable diseases. To confront this concern, healthcare providers need to address the reasons for inactivity. Unfortunately, limited tools exist to address barriers of physical activity (PA). The purpose of our study was to develop and psychometrically evaluate a PA barrier scale for adults 50 years and older. The scale is called the Inventory of Physical Activity Barriers (IPAB) and it was developed, refined, and evaluated using a cross-sectional and a modified Delphi study. We had two groups of participants: 39 adults (50 years and older) provided survey pilot data for psychometric evaluation, and nine interprofessional PA experts assisted with finalizing the scale. Participants completed a demographic questionnaire, Physical Activity Vital Sign questionnaire, and IPAB. The IPAB’s refinement was guided by item-scale correlations, descriptive statistics, and consensus among the PA experts. Construct validity was examined by comparing mean IPAB scores of inactive and active participants via independent t-test. Internal consistency was assessed via Cronbach Alpha. The IPAB was refined from 172 items to 40 items and found to be internally consistent (α=.97) and able to differentiate individuals who do and do not meet the recommended 150 minutes of weekly PA (p=0.01). These preliminary results show the IPAB is a reliable assessment of PA barriers for adults 50 years and older, and are promising for the scale’s construct validity and support further psychometric evaluation of the tool.

